# Identification by MALDI-TOF MS and Antibiotic Resistance of *Riemerella anatipestifer*, Isolated from a Clinical Case in Commercial Broiler Chickens

**DOI:** 10.3390/vetsci8020029

**Published:** 2021-02-17

**Authors:** Athina Tzora, Stylianos Skoufos, Eleftherios Bonos, Konstantina Fotou, Achilleas Karamoutsios, Aikaterini Nelli, Ilias Giannenas, Anastasios Tsinas, Ioannis Skoufos

**Affiliations:** 1 Laboratory of Animal Health, Food Hygiene and Quality, Department of Agriculture, School of Agriculture, University of Ioannina, Kostakioi Artas, 47100 Arta, Greece; steliosskoufos@gmail.com (S.S.); ebonos@uoi.gr (E.B.); kfotou@uoi.gr (K.F.); a.karamoutsios@uoi.gr (A.K.); k.nelli@uoi.gr (A.N.); actsinas@uoi.gr (A.T.); jskoufos@uoi.gr (I.S.); 2 Laboratory of Nutrition, School of Veterinary Medicine, Faculty of Health Sciences, Aristotle University of Thessaloniki, 54124 Thessaloniki, Greece; igiannenas@vet.auth.gr

**Keywords:** *Riemerella anatipestifer*, broiler chickens, MALDI-TOF, antibiotic resistance, clinical disease, bacteria

## Abstract

The Gram-negative bacterium *Riemerella anatipestifer* (RA) is known to cause clinical disease with severe economic impacts primarily in ducks and less frequently in geese and turkeys. RA was isolated and identified in broiler chickens, from a clinical case in a commercial broiler farm located in the southwest mainland of Greece. The morbidity and the mortality in the broiler house were estimated at 10% and 5% respectively. The observed clinical signs appeared at the age of 30 to 42 days with respiratory distress (dyspnea), white fluid feces and stunting. Post-mortem examinations displayed serositis, pericarditis, perihepatitis and airsacculitis. Edematous swelling around the tibio-tarsal joints was observed in some birds. Tissue samples from lesions were streaked on selective media. Three bacterial isolates were identified by matrix-assisted laser desorption/ionization time-of-flight mass spectrometry (MALDI-TOF MS). Moreover, an antibiogram analysis was performed for the three RA strains, using a pattern of 16 common antibiotics to advocate the most effective drugs for a proper treatment. All the RA isolates were sensitive to ceftiofur, sulphamethoxazole–trimethoprim and amoxicillin, whereas all were resistant to gentamicin, tylosin, tetracyclin, colistin sulphate, spectinomycin, lincomycin and oxytetracycline.

## 1. Introduction

*Riemerella anatipestifer* (RA) is the aetiologic agent for a serious contagious bacterial disease of the avian species that causes substantial economic losses in commercially important poultry worldwide [1]. RA is a Gram-negative, non-motile, non-spore forming and rod-shaped bacterium belonging to the family Flavobacteriaceae of the phylum Bacteroidetes [2]. The disease occurs worldwide as an acute or chronic septicemia and has been recognized as a prominent problem in countries that have intensive duck production [3].

Outbreaks of RA have been reported for domestic ducks and turkeys, the first ones resulting in a high mortality rate of up to 75% and a morbidity that is usually as high as 100% [1,4,5,6]. Furthermore, within the wild birds, some cases of the disease have also been mentioned [7,8]. In general, affected birds have displayed clinical symptoms such as ocular and nasal discharges, mild coughing and sneezing, tremors of the head and neck, joints’ inflammation causing a paddling movement of the legs. Post-mortem lesions were commonly characterized by fibrinous polyserositis, pericarditis, perihepatitis, airsacculitis and in chronic cases by salpingitis and meningitis [9].

The present study describes an identified outbreak of RA disease in broiler chickens in Greece, including clinical signs and post-mortem lesions. For the first time, three clinical isolates of RA in broiler chickens were identified and differentiated by matrix-assisted laser desorption ionization-time-of-flight mass spectrometry (MALDI-TOF MS). In addition, the antimicrobial sensitivity of the isolated RA strains was examined, in order to advocate the best drugs for treatment.

## 2. Materials and Methods

### 2.1. Ethical Statement

All birds used in this study were collected by veterinarians from a clinical case of disease in a commercial broiler chicken farm. These birds were humanely euthanized due to severe clinical illness and stunted growth and submitted to post-mortem examination according to national regulations.

### 2.2. Selections of Cases

In October 2019, a possible outbreak of a contagious respiratory disease was reported in single house with 20,000 broilers, from a commercial broiler chicken farm with a total capacity for 70,000 broilers, located in the southwest area of Greece. Based on the farm data, the morbidity in this broiler house was estimated at approximately 10% and mortality at 5%. Under veterinary supervision, a combination of doxycycline, tylosin and colistin was administered to the birds via drinking water as blank treatment, since the symptoms were suggestive of colibacillosis.

From this farm, six live birds (40-days-old, Ross 308) were submitted for examination to the Laboratory of Animal Health—Food Hygiene and Quality of the School of Agriculture, University of Ioannina. All birds were characterized by stunted growth, estimated 30–40% lower weight, in comparison to the average flock weight as well as to the target weights for their age [10]. Clinical examination showed that out of the six birds, five were presenting clinical symptoms such as respiratory distress (dyspnea) and white fluid feces.

### 2.3. Post-Mortem Examination

Post-mortem examination was performed to evaluate the severity of organ damage. From all six birds, tissue samples were collected from viscera with profound lesions, including liver, air sacs and pericardium. 

### 2.4. Riemerella Anatipestifer Isolation and Identification

All tissue samples were immediately streaked on Columbia sheep blood (CSB) agar, containing 5% sheep blood, and also on McConkey agar. 

Initially, CSB were incubated aerobically for 24 h at 37 °C. However, after the identification of RA by the MALDI-TOF MS (as described below) in some CSB agars, sub cultured colonies were taken and streaked again in CSB agars and incubated for 24 h at 37 °C under microaerophilic conditions (5% CO_2_). The same samples that have been inoculated on McConkey agar plates and incubated aerobically at 37 °C, did not show any bacterial growth 24 or 48 h later. 

The phenotypic identification of the isolates was based on their morphology, culture characteristics and biochemical tests. The isolates showed non hemolytic zone on blood agar plate. Preliminary identification was based on phenotypic characteristics (growth, shape, motility). Smears were stained with Gram’s stain according to standard techniques. Gram’s staining revealed the presence of single or paired Gram-negative short rods. In addition, catalase, oxidase and gelatinase tests were performed [11,12,13]. The physiologic and biochemical properties were compared to those of type strain ATCC 11845. All agars and reagents were procured from Merck KGaA (Darmstadt, Germany).

### 2.5. Sample Preparation for MALDI-TOF MS Identification: Parameters and Main Spectral Profile (MSP) Dendrogram Construction

The identification of RA isolates was conducted using the mass spectrometer Microflex LT MALDI-TOF MS (Bruker Daltonic, Bremen, Germany). A total of 240 laser shots in 40 shot steps were summarized and each spot was measured twice automatically with AutoXecute acquisition control software (flexControl 3.4; Bruker Daltonic, Bremen, Germany). Ethanol-formic acid-acetonitrile standard operation protocol, was evaluated in order to achieve a complete protein extraction [14]. The reference library from Bruker Daltonic was used to identify the isolates. The instrument was calibrated using a Bacterial Test Standard (BTS-containing a typical *Escherichia coli* DH5 alpha peptide and protein profile plus additional proteins) (Bruker Daltonic, Bremen, Germany). The generated peak list was matched against the database using the pattern-matching algorithm of MALDI Biotyper software version 3.4 (Bruker Daltonic, Bremen, Germany). Twenty four mass spectra of each RA strain were obtained and were converted into master spectra (MSPs). Five RA reference strains databank entries were included as controls (MALDI Biotyper 3.4, Bruker Daltonic, Bremen, Germany).

### 2.6. Antibiotic Sensitivity Test

Antibiotic sensitivity pattern of three RA isolates was carried out using the standard Kirby–Bauer disk diffusion method [15]. Sixteen antibiotics were used to determine the antibiotic susceptibility of RA isolates, including: 25 μg of Amoxicillin (AMOXY25, Oxoid Basingstoke, Hampshire, UK), 10 μg of Ampicillin (AMP10, Oxoid, Basingstoke, Hampshire, UK), 30 μg of Ceftiofur (CFT30, Rosco Diagnostica, Taasrup, Denmark), 30 μg of Ciprofloxacin (CIP30, Oxoid, Basingstoke, Hampshire, UK), 10 μg of Colistin sulfate (CT10, Oxoid, Basingstoke, Hampshire, UK), 30 μg of Doxycycline (DOXYC30, Rosco Diagnostica, Taasrup, Denmark), 5 μg of Enrofloxacin (ENR5, Oxoid, Basingstoke, Hampshire, UK), 10 μg of Gentamicin (CN10, Oxoid, Basingstoke, Hampshire, UK), 15 μg of Lincomycin (MY15, Oxoid, Basingstoke, Hampshire, UK), 10 μg of Neomycin (N10, Oxoid, Basingstoke, Hampshire, UK), 30 μg of Oxytetracycline (OT30, Sanofi Diagnostics Pasteur, Marnes-la-Coquette, France), 10 μg of Penicillin G (P10, Oxoid, Basingstoke, Hampshire, UK), 100 μg of Spectinomycin (SH100, Oxoid, Basingstoke, Hampshire, UK), 25 μg of Sulphamethoxazole–Trimethoprim (SXT25, Oxoid, Basingstoke, Hampshire, UK), 30 μg of Tetracyclin (TE30, Oxoid, Basingstoke, Hampshire, UK) and 30 μg of Tylosin (TY30, Liofilchem, Teramo, Italy). Disk diffusion analysis was performed on Mueller–Hinton agar enriched with 5% defibrinated sheep blood and was incubated at 37 °C in microaerophilic atmosphere with 5% CO_2_. Quality control isolates included *Escherichia coli* ATCC 25,922 and *Staphylococcus aureus* ATCC 25923. Inhibitory zone diameters were measured after 24 h of incubation. 

The resistance breakpoints were interpreted according to the criteria provided by Clinical and Laboratory Standards Institute (CLSI) documents M100-S21 and VET01S [16,17], of the National Food Chain Safety Office, Veterinary Diagnostic Directorate. The interpretation was based on CLSI document VET01 fifth edition [17], as well as described in previous reports [18,19].

## 3. Results

### 3.1. Post-Mortem Findings

**Figure 1:**
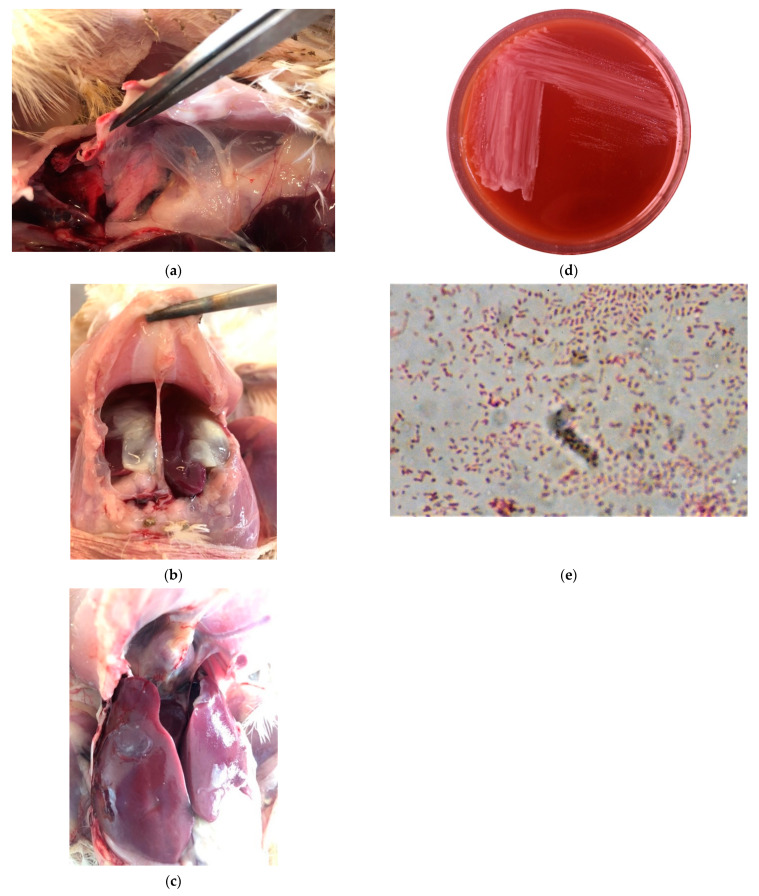
*Riemerella anatipestifer*: (**a**) Airsacculitis of the thoracic air sacs in broiler chicken filled with organized yellow casts; (**b**) Serositis in broiler chicken; (**c**) Pericarditis & perihepatitis in broiler chicken; (**d**) Transparent, glistening and non-hemolytic colonies on Sheep Blood Agar (CBS); (**e**) Gram-stained smear from culture showing the typical pleomorphic appearance.

Post-mortem lesions were observed in five of the six examined birds. The examinations showed airsacculitis, serositis, pericarditis and perihepatitis (Figure 1A–C). Edematous swelling around the tibio-tarsal joints was observed in three out of the six birds. 

### 3.2. Isolation and Identification of Riemerella anatipestifer

Streaked organ samples on CSB agar were positive for RA in two out of five affected birds. Positive samples were designated as: RA1 (Sample from air sac from bird No.3), RA2 (Sample from liver from bird No.5) and RA3 (Sample from pericardium from bird No.5). The results of the biochemical tests for RA as shown in Table 1. Catalase Oxidase and Gelatinase tests were positive of all three isolates. The physiologic and biochemical properties were similar to those of type strain ATCC 11845.

Typical individual colonies of each isolate were picked for MALDI-TOF analysis. These isolates were identified as RA by MALDI-TOF MS, using the reference database (Table 1). The log score of RA2 (2.15) indicating “secure genus identification, probable species identification,” whereas the log scores of RA1 (2.30) and RA3 (2.35) indicating “high probable species identification,” based on the reference database guidelines.

Although RA isolates (RA1, RA2 and RA3) were grown under aerobic conditions on CSB agar initially, the growth of these bacteria was optimal in the same medium under an atmosphere of 5% CO_2_. Colonies were small, 1–2 mm in diameter, transparent, glistening and non-hemolytic (Figure 1D). There was no growth on MacConkey agar. Microscopic observation of CSB agar colonies revealed short, Gram-staining-negative, non-sporulating, rod-shaped bacteria (Figure 1E). 

**Figure 2:**
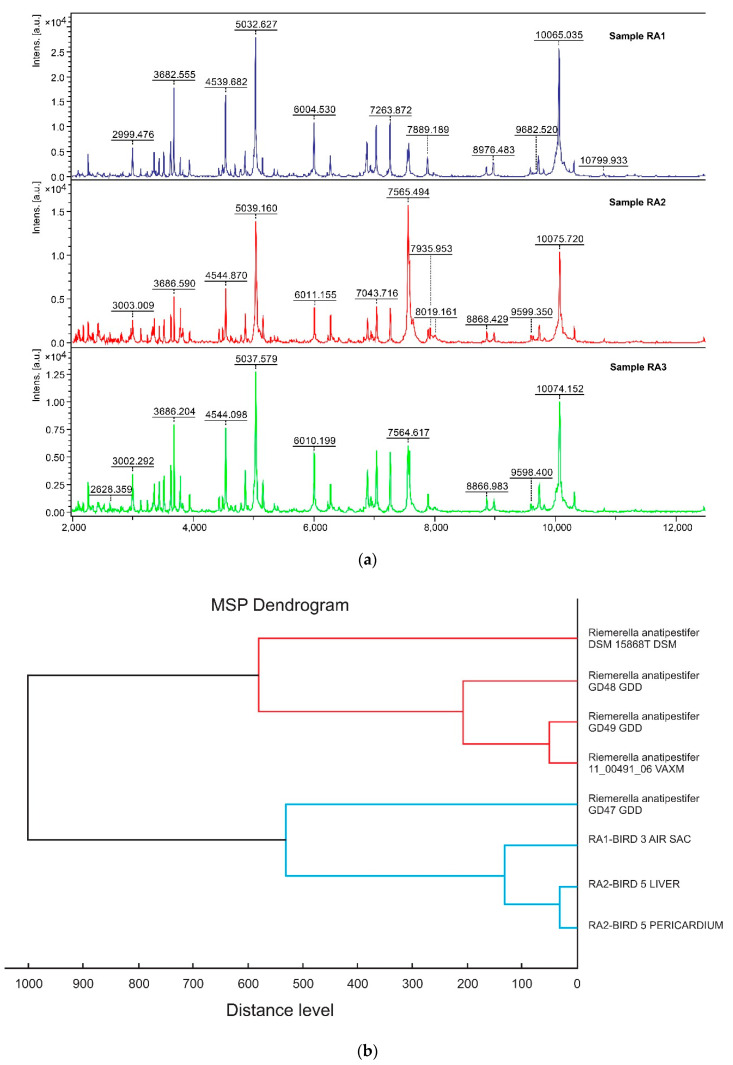
(**a**) Mass spectra for *Riemerella anatipestifer (RA)* isolates, analyzed by MALDI-TOF MS; (**b**) MSP dendrogram: Classification of three isolates of *Riemerella anatipestifer*, based on the protein mass patterns, analyzed by MALDI Biotyper software using MALDI-TOF MS.

For the construction of the MSP dendrogram based on the results from MALDI-TOF MS (Figure 2A), the data were processed with default software settings. The resulting MSP dendrogram (Figure 2B) shows that the RA2 and RA3 isolates of RA are closely related (distance level < 100) and clustered with the RA1 strain (distance level < 200) to the species level [20].

### 3.3. Antibiotic Susceptibility Testing Results

Table 2 presents the antibiotic sensitivity pattern of RA isolates and the resistance breakpoints of the antibiotics used in this study. The three RA isolates displayed a clear sensitivity profile to three antibiotics, which were ceftiofur, sulphamethoxazole–trimethoprim and amoxicillin. All the RA isolates displayed an expanding resistance pattern to gentamicin, tylosin, tetracyclin, colistin sulfate, spectinomycin, lincomycin and oxytetracycline. Other antibiotics which were tested such as ampicillin, penicillin, enrofloxacin, neomycin, ciprofloxacin and doxycycline, showed variable resistance and sensitivity pattern between the clinical RA isolates.

## 4. Discussion

Our results document a detected clinical case of an infection caused by RA in a commercial poultry farm in Greece. Based on available published literature, for the first time, in our case report, clinical RA isolates originating from broiler chickens were identified at species level, by MALDI-TOF MS.

In the published literature clinical cases of RA in chickens are scarce. Rosenfeld [21] reported that RA had been isolated and identified in chickens with atypical signs. Since then, a field outbreak of RA disease in chickens has also been described in China [22].

In our clinical and post-mortem findings, the observed post-mortem gross lesions included fibrinous exudate in the pericardial cavity and over the surface of the liver, as well as airsacculitis filled with organized yellow casts, all of which are characteristic findings reported in avian species [8,23,24,25]. 

Standard methods of isolating microorganisms in suitable agar media were initially used, with samples taken from post-mortem lesions from the pericardium, airsacs and liver. Out of all samples from six birds, RA growth was detected only in three plated organ samples from two birds. We identified these colonies as RA using the MALDI-TOF MS, allowing us to continue their subcultures under microaerophilic conditions with much better growth. At the same time, no other of the possible examined pathogenic bacteria like *E. coli* and *Salmonella* spp. were grown from the same tissues. These observations are in line with previous reports in geese [23].

Diagnosis of RA infection is difficult, using standard microbiological procedures [13,26]. Particularly in our case both CSB and MacConkey agar were inoculated from the lesions found in the examined birds. RA colonies were grown only on the CSB agar, confirming the existence of this bacterium. Additionally, the microaerophilic conditions during the second cultivation promoted the growth of RA and revealed its existence. Polymerase chain reaction (PCR) methods have been used by many studies to identify the isolates obtained but most of the PCR assays designed for RA detection, are proven to be ineffective to detect or specify all RA strains [27,28]. The use of a MALDI Biotyper is considered to give more reliable results for RA identification, making use of well-described RA reference strains [23,26,29], while being adequately simple and cost-effective for routine laboratory use [23]. RA have been successfully isolated and identified with MALDI-TOF MS from clinical cases from ducks, goose and turkeys, as previously described [23,26,29]. 

In our study, clinical RA isolates originating from broiler chickens were identified at species level, by MALDI-TOF MS. The protein profiling, via MALDI-TOF MS, of the three RA clinical isolates indicated that the two RA isolates from different tissues of the same bird (RA2-liver & RA3-pericardium of bird No. 5) are clustered together with high affinity (distance level < 100) and subclustered with the RA1 isolate from the other infected bird (RA1-airsac of bird No. 3) (distance level < 200) indicating high correlation. All the Bruker databank entries of RA, except from the databank entry “*Riemerella Anatispetifer* 11-00491-06 VAXM,” have been derived from ducks. Our three RA clinical isolates derived from chickens, are not reliable related (distance level > 500) [20], with these RA Bruker databank entries (including the RA reference type strain (DSM 15868T R). In the resulting MSP dendrogram we observed that the RA clinical isolates have been reliably classified to the same species and to different subspecies, which demonstrates the existence of the genetic variation into RA strains [30].

The large genetic diversity of the RA strains with the poor cross-protection between them discourages the prevention using vaccines, making antimicrobial therapy the primary method of fighting RA infection [31]. Thus, in vitro drug sensitivity testing is essential for the selection of an appropriate antibiotic for a given situation. 

The severity of RA infections in the avian breeding industry, has mainly been controlled by the wide use of quinolones, tetracyclines and cephalosporins, which have consequently led to the emergence of antibiotic-resistant strains specifically in ducks [32]. Likewise, with many other bacterial pathogens worldwide, the incidence of drug resistance in the treatment of RA infections is increasing [33,34]. 

In our study, three RA isolates were tested versus 16 antibiotics, widely used agents in poultry industry, in order to determine the susceptibility in each antimicrobial agent. All three RA isolates displayed a clear sensitivity profile to amoxicillin, ceftiofur and sulphamethoxazole-trimethoprim. Chang et al. [35] reported a high percentage (97.4%) of the tested RA isolates which were susceptible to amoxicillin. Ceftiofur, is an approved third generation cefalosporin for food animal use in the United States and Europe [36]. Studies in duck isolates in Taiwan also approved that the ceftiofur is among the most effective antibiotics. Chang et al. [35] found that sulphamethoxazole-trimethoprim showed 57% and 50% efficacy RA isolates from geese and ducks, respectively. Sun et al. [37] noted that Chinese ducks’ RA isolates, were resistant to sulphonamide, while 75% of the strains were susceptible to sulphamethoxazole-trimethoprim. 

According to our results, all the RA isolates displayed an expanding resistance pattern to eight antibiotics: colistin sulfate, spectinomycin, gentamicin, lincomycin, neomycin, oxytetracycline, spectinomycin, tetracycline and tylosin. Chang et al. [35] examined antibiotic resistant of RA in ducks and geese, demonstrated that 50% or more of the isolates had developed resistance against commonly used antibiotics. Gyuris et al. [19] found that more than one half of the RA isolates from geese and ducks in Hungary had developed resistance against gentamicin. The high prevalence of colistin resistance in the isolates was also reported in previous studies [35,38]. According to the same results, more than 70% of the isolates were resistant to lincomycin as well [35,39]. Colistin and lincomycin are approved for use in feed treatments for bacterial infections in poultry. Apart from colistin, resistance has also emerged to spectinomycin, which is often used in poultry practice [19].

In this study, differences were detected in the sensitivity and resistance pattern between the three RA isolates, in several antibiotics such as ampicillin, penicillin, enrofloxacin, neomycin, ciprofloxacin and doxycycline. Of remarkable note is also the difference in sensitivity and resistance at doxycycline, an antibiotic commonly used in poultry production. In a previous study, conducted by Zhong et al. [18] it was reported that RA isolates were resistant to ampicillin, which is in agreement with our results as two from the three RA isolates were resistant to this drug. Two of the three RA isolates in this study were resistant to enrofloxacin. Sensitivity to enrofloxacin against RA isolated from ducklings was reported by Turbahn et al. [40] and Soman et al. [41]. 

Regarding the origin of the contamination, our assumption was that the pathogen possibly spread from a duck breeding farm that was adjacent to the broiler farm, where during the same period faced some moderate mortalities from unspecified causes as it was reported by the farmer. Unfortunately, it was not possible to procure samples from these ducks for laboratory analysis. Consequently, it is very important to highlight the need to maintain a high level of biosecurity and good hygiene standards and practices, especially in areas with high numbers of animal and multiple species of reared poultry.

## Figures and Tables

**Table 1:** Identification of *Riemerella anatipestifer* from broiler chicken tissues using culture, staining and biochemical tests and matrix-assisted laser desorption/ionization time-of-flight mass spectrometry (MALDI-TOF MS) analysis based on the Bruker Daltonic database.

Culture Technique and Biochemical Tests	RA1 (Bird No.3- Air Sac)	RA2 (Bird No.5- Liver)	RA3 (Bird No.5- Pericardium)
Growth on Columbia sheep blood (CSB) agar aerobically	+	+	+
Hemolysis on CSB agar	-	-	-
Growth on CSB microaerophilically	+	+	+
Growth on MacConkey agar	-	-	-
Gram’s reaction	-	-	-
Catalase test	+	+	+
Oxidase test	+	+	+
Gelatinase test	+	+	+
**MALDI-TOF MS analysis**			
Matched pattern on the Bruker Daltonic database	*R. anatipestifer* GD 47 GDD	*R. anatipestifer* GD 47 GDD	*R. anatipestifer* GD 47 GDD
Log (score) value *	2.30	2.15	2.35

* According to MALDI Biotyper software version 3.4 (Bruker Daltonic, Bremen, Germany) a log score value between 2.0–3.0 corresponds to “high confidence species identification”.

**Table 2:** Resistance breakpoints and antibiotic sensitivity pattern of *Riemerella anatipestifer* from broiler chicken tissues.

	Interpretive Criteria: Zone Diameter (mm)			RA1 (Bird No.3- Air Sac)	RA2 (Bird No.5- Liver)	RA3 (Bird No.5- Pericardium)
	Resistant	Intermediate	Susceptible			
Amoxycillin (25 μg)	≤18	19–20	≥21	Sensitive	Sensitive	Sensitive
Ampicillin (10 μg)	≤23		≥24	Resistant	Resistant	Sensitive
Ceftiofur (30 μg)	<18		≥21	Sensitive	Sensitive	Sensitive
Ciprofloxacin (30 μg)	≤15	16–20	≥21	Sensitive	Sensitive	Intermediate
Colistin Sulphate (10 μg)	≤16	17–19	≥20	Resistant	Resistant	Resistant
Doxycycline (30 μg)	<22		≥23	Resistant	Sensitive	Sensitive
Enrofloxacin (5 μg)	≤16	17–22	≥23	Resistant	Resistant	Sensitive
Gentamicin (10 μg)	≤12	13–14	≥15	Resistant	Resistant	Resistant
Lincomycin (15 μg)	<17		≥21	Resistant	Resistant	Resistant
Neomycin (10 μg)	≤16		≥17	Resistant	Sensitive	Resistant
Oxytetracycline (30 μg)	<17		≥19	Resistant	Resistant	Resistant
Penicillin G (10 μg)	≤23		≥24	Sensitive	Sensitive	Resistant
Spectinomycin (100 μg)	≤15	16	≥17	Resistant	Resistant	Resistant
Sulphamethoxazole-Trimethoprim SΧΤ (25 μg)	≤10	11–15	≥16	Sensitive	Sensitive	Sensitive
Tetracycline (30 μg)	≤22		≥23	Resistant	Resistant	Resistant
Tylosin (30 μg)	<14		≥18	Resistant	Resistant	Resistant

## Data Availability

The data presented in this study are available on request from the corresponding author.
